# A Study to Identify Medication-Related Problems and Associated Cost Avoidance by Community Pharmacists during a Comprehensive Medication Review in Patients One Week Post Hospitalization

**DOI:** 10.3390/pharmacy7020051

**Published:** 2019-05-29

**Authors:** Roxane L. Took, Yifei Liu, Peggy G. Kuehl

**Affiliations:** 1 Division of Pharmacy Practice, St. Louis College of Pharmacy, St. Louis, MO 63110, USA; roxane.took@stlcop.edu; 2 Division of Pharmacy Practice and Administration, School of Pharmacy, University of Missouri-Kansas City, Kansas City, MO 64108, USA; kuehlp@umkc.edu

**Keywords:** community pharmacy, medication therapy management, medication-related problems, medication discrepancies, continuity of patient care, cost avoidance

## Abstract

**Objectives:** To determine the numbers of medication discrepancies and medication-related problems (MRPs) identified and resolved when providing a transitions of care comprehensive medication review (CMR) after hospital discharge within a community pharmacy; and to estimate the cost-avoidance value of this service. **Methods:** Community pharmacists provided CMRs to covered employees and dependents of a self-insured regional grocery store chain who were discharged from the hospital. Data was collected prospectively over 4 months. Discrepancies were identified among patients’ medication regimens by comparing the hospital discharge record, the pharmacy profile, and what the patient reported taking. MRPs were categorized into ten categories, as defined by the OutcomesMTM^®^ Encounter Worksheet. Interventions were categorized using the severity scale developed by OutcomesMTM^®^, a Cardinal Health company. Data were analyzed using descriptive statistics and bivariate correlations. **Results:** Nineteen patients were enrolled in the program. Pharmacists identified 34 MRPs and 81 medication discrepancies, 1.8 and 4.3 per patient, respectively. The most common type of MRP was underuse of medication (70.6%). Significant positive correlations were found between the number of scheduled prescription medications and the number of medications with discrepancies (p ≤ 0.01; r = 0.825) and number of scheduled prescription medications and the number of MRPs (p ≤ 0.01; r = 0.697). Most commonly, the severity levels associated with the MRPs involved the prevention of physician office visits or addition of new prescription medications (n = 10 each); however, four emergency room visits and three hospitalizations were also avoided. The total estimated cost avoidance was $92,143, or $4850 per patient. Extrapolated annual cost savings related to this service would be $276,428. **Conclusions:** This transitions of care service was successful in identifying and addressing MRPs and discrepancies for this patient population. By providing this service, community pharmacists were able to prevent outcomes of various severities and to avoid patient care costs.

## 1. Introduction

Medication errors resulted in an estimated 251,454 hospital deaths in 2013 [1]. It is estimated that $17 to $29 billion is spent on preventable adverse events annually. In addition, up to 80% of serious medical errors involve miscommunication among medical providers [2]. Given the high prevalence, morbidity, and cost of such errors, much attention has been drawn to discrepancies and medication-related problems (MRPs) that occur during transitions of care, a time when patient care is handed off among inpatient and outpatient providers.

Previous research has shown the value of pharmacist involvement in transitions of care. Inclusion of a pharmacist has lowered rates of re-hospitalizations and emergency department visits, improved patient satisfaction with care, and increased medication adherence [3,4]. Many hospitals and pharmacy directors have also recognized the importance of involving pharmacists in transitions of care activities for hospitalized patients [5].

Community pharmacists have aided in the transition process by calling patients within 2 to 7 days after discharge to reinforce the discharge plan, provide patient education, address any medication-related issues, and review medications [6,7,8,9]. The extent to which pharmacists have been involved has varied from a brief phone call to an extensive face-to-face visit with a pharmacist and 2-week follow-up [6,7,8,9,10]. For instance, in the study of TransitionRx, community pharmacists performed face-to-face visits with patients admitted for congestive heart failure, chronic obstructive pulmonary disease, and pneumonia [10]. These visits consisted of medication reconciliation, comprehensive medication review (CMR), disease state education, patient counseling on new medications, and self-management education. Patients were provided with a personal medication list (PML), medication action plan (MAP), self-monitoring logs, and educational materials. Physicians were provided with a summary of this face-to-face visit and any MRPs or discrepancies were resolved. There was a readmission rate of 7% for patients who met with a pharmacist vs. 20% in the usual care group.

Kelling et al. described the implementation of medication therapy management (MTM)-based transitions of care with a Medicaid population in a grocery store setting [11]. In the study, 17 Medicaid patients met face-to-face with a pharmacist in either a supermarket chain pharmacy or a local federally qualified health center. Patients were asked to bring all prescription and non-prescription medications along with discharge paperwork. Pharmacists completed a medication reconciliation using the discharge paperwork and resolved any MRPs or side effects via phone or fax. Patients were mailed a PML and MAP. Reimbursement was provided through Medicaid, and additional payments for each MRP were identified, resolved, and entered into the OutcomesMTM^^®^^ Connect™ Platform. For these 17 patients, 50 pharmacologic recommendations and 36 behavior modification recommendations were advised.

While these studies demonstrated that pharmacists could identify and resolve MRPs during transitions of care, none provided an estimate of the cost avoidance of these interactions or examined the severity of MRPs involved. Therefore, the objectives of this study were to determine the numbers of medication discrepancies and MRPs identified and resolved when community pharmacists performed a CMR for employees and dependents of a self-insured regional grocery store/pharmacy chain following hospital discharge; and to associate each MRP with cost-avoidance to quantify the value of this service.

## 2. Service Description

Balls Food Stores (BFS) is a self-insured regional grocery store and pharmacy chain with approximately 2600 covered lives under their health insurance plan. The company provides many BFS pharmacist-led employee health programs and patient services, including one-on-one disease state management visits for employees and dependents with uncontrolled hypertension, dyslipidemia, diabetes mellitus, and history of myocardial infarction or stroke; tobacco cessation classes for employees and dependents; immunizations including travel vaccine clinics; MTM services; and health screenings are also conducted. BFS implemented a transitions of care service in the fall of 2013 to provide transitions of care for covered employees and dependents. Three clinical pharmacists, employed by BFS, performed this service.

Using information provided by BFS’ insurance plan administrator, pharmacists were able to identify covered employees and dependents discharged from a hospital or long-term care facility. Patients were eligible for this service if they were ≥18 years old, insured by BFS, and hospitalized for at least two consecutive days. Patients were not eligible for this service if hospitalized for childbirth, suicidal attempts, or psychiatric conditions. Eligible patients were assigned to one of three pharmacist providers who contacted these patients within 7 days of discharge to schedule a one-on-one CMR. These providers were clinical pharmacists who had residency-training or were undergoing residency training. Visits were completed as soon as the patient and pharmacist were available and performed either in-person or via telephone. Pharmacists asked patients to provide discharge papers, all prescription medications, over-the-counter products, herbal products, and supplements for the CMR visit. If the patient did not have their discharge paperwork, the pharmacist requested a copy from the medical records department of the discharging hospital. These visits were 30 to 60 min long and were documented using a modified CMR worksheet and the OutcomesMTM^^®^^ Encounter Worksheet (see Supplementary Materials). Pharmacists compared the patient’s current medications to the discharge orders and the active medications in the pharmacy dispensing profile. Pharmacists were able to determine the number of MRPs detected and categorize them using the “drug therapy problem detected” section of the OutcomesMTM^^®^^ Encounter Worksheet. When an MRP was identified, pharmacists took action to resolve it either directly with the patient or by communicating with the patient’s health care provider.

Within 7 days of CMR completion, patients were mailed a PML, MAP, and cover letter with the pharmacist’s hours of availability and contact information. Pharmacists completed documentation for each MRP identified and followed up with each patient 1 week after mailing/delivering the medication list to ensure that the patient received the letter and understood the action items required to resolve the MRP.

## 3. Methods

For this research, data for all patients seen by this service from 1 December 2013 to 31 March 2014 were included for analysis. All MRPs and medication discrepancies were transcribed from the patient care chart and recorded onto a master data collection sheet. Discrepancies were identified by comparing the pharmacy dispensing profile, discharge paperwork, and patient interview. MRPs were categorized into 10 categories, as defined by the OutcomesMTM^^®^^ Encounter Worksheet and discrepancies were assigned to one of three categories: Discrepancy between discharge instructions and pharmacy medication profile, discrepancy between discharge instructions and patient self-report, and discrepancy between pharmacy medication profile and patient self-report.

After completion of a premium subscription agreement and data use agreement with OutcomesMTM^^®^^, encounters were entered into the OutcomesMTM^^®^^ Connect™ Platform [12], and severity levels were reviewed and validated by OutcomesMTM^^®^^ staff. OutcomesMTM^^®^^ is an MTM administrative service company that allows pharmacists to bill for MTM services through their electronic platform. This platform has been used primarily by community pharmacies to document CMRs and targeted interventions (TIPs). OutcomesMTM^^®^^ can also be used to identify cost savings associated with pharmacist’s interventions to resolve therapy and adherence problems.

Each MRP was assigned a severity level and cost avoidance was assigned to each intervention using the OutcomesMTM Actuarial Investment Model^^®^^ [13]. All data were stored in accordance with applicable federal privacy regulations. The methodology of OutcomesMTM^^®^^ was used to document severity level and cost savings associated with resolution of MRPs. MRPs included: Needs additional drug therapy, unnecessary drug therapy, suboptimal drug, dose too low, adverse drug reaction, drug interaction, dose too high, overuse of medication, underuse of medication, and inappropriate administration or technique. Variables collected included patient demographics, types of discrepancies, types of MRPs, severity levels, number of physicians per patient, and number of prescriptions. Data were analyzed using descriptive statistics and bivariate correlations. A *p* value ≤ 0.05 was regarded as statistically significant. This study was approved by the University of Missouri—Kansas City Adult Health Sciences Institutional Review Board.

## 4. Results

**Figure 1:**
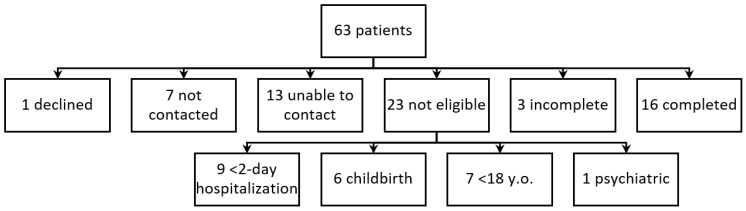
Flowchart of patients included and excluded from the service.

For the 4 months during which the transitions of care service was studied, 63 patients were discharged from a hospital or intermediate care facility, and 40 met the inclusion criteria. Figure 1 represents the patients included and excluded from the service. Because of initial issues with reporting from the insurance administrator, seven patients were not identified as eligible for this service until after 7 days post-discharge. These patients were not contacted for this service. Pharmacists attempted to reach 13 patients three or more times regarding this service with no response; therefore, these patients could not be included in the service. One eligible patient declined to participate. All 19 participating patients received a CMR and either a complete or incomplete medication reconciliation. Sixteen patients completed all aspects of the service (CMR, provision of discharge paper work, and medication reconciliation). For three patients, pharmacists were unable to obtain discharge paperwork from the hospital or the patient and thus had an incomplete medication reconciliation. For the 19 participating patients, the majority were male (68%), and the mean age was 60 (range 46 to 70) years old. Patients took an average of six (range 2 to 16) scheduled medications daily for their reported disease states (mean 4.4 range 1 to 16). Patients were hospitalized for a mean of 5 (range 2 to 12) days. This service was primarily provided via phone versus face-to-face by request of the patient. Community pharmacists identified and resolved 34 MRPs and 81 medication discrepancies after completing all aspects of the service. On average, 4.3 discrepancies and 1.8 MRPs were identified per patient. The frequency of MRPs is listed in Table 1. The most common type of MRP was underuse of medication (70.6%). Medication discrepancies were similarly distributed among the three categories (31 discrepancies between discharge paperwork and patient-self report, 25 discrepancies between discharge instructions and pharmacy dispensing profile, and 25 discrepancies between pharmacy dispensing profile and patient-self report), with a slightly higher percentage identified in the category of discrepancy between discharge instructions and patient self-report.

The two most commonly observed severity levels identified in the OutcomesMTM^^®^^ platform were “prevented physician visit” and “prevented additional prescription order”. Severity levels and frequency for each MRP are listed in Table 2. In addition to interventions that prevented additional prescription orders or physician visits, the pharmacists were able to prevent four emergency room visits and three hospital admissions. For example, during two medication reviews and consultations, the pharmacists found that the patient was prescribed a phosphodiesterase inhibitor and nitroglycerin. The pharmacist provided education to each of these patients about the risks of taking these medications concurrently and was able to resolve this issue after consulting the patient’s primary care physician. OutcomesMTM^^®^^ staff determined that the pharmacist’s intervention in these visits prevented an emergency room visit due to the risk for significant hypotension.

The OutcomesMTM Actuarial Investment Model^^®^^ determined that the MRPs resolved by pharmacists over the period of 4 months provided a total estimated cost avoidance of $92,143, or $4850 per patient. If this rate of MRPs were to continue over a 12-month period, the annual cost savings related to this service would have been $276,428.

There was a statistically significant positive correlation between the number of scheduled prescription medications and the number of medications with discrepancies (*p* ≤ 0.01; r = 0.825); and the number of scheduled prescription medications and the number of MRPs (*p* ≤ 0.01; r = 0.697) (Table 3). In addition, statistically significant positive correlations were found between the following variables: The number of scheduled medications and the number of disease states, the number of medications with discrepancies and each of the following variables: Number of MRPs, inappropriate adherence, needing an additional drug therapy, and the number of medications with each of the following variables: Number of discrepancies, number of MRPs, and severity level determined by OutcomesMTM^^®^^. There was a strong negative correlation between patients who had a medication reconciliation completed in the hospital prior to discharge and the number of medications that were found with discrepancies after discharge (*p* ≤ 0.01; r = −0.667).

## 5. Discussion

BFS is a self-insured company that was interested to know whether this pilot program would reduce costs associated with transitions of care for covered employees and dependents, and whether this program should be continued. Previous research has shown positive benefits for transitions of care services from the hospital setting, but limited research has been completed in the community setting.

Our study found a strong negative correlation between patients who received prior discharge counseling in the hospital and the number of medications with discrepancies. This is consistent with previous findings. For example, an urban, academic, safety-net hospital created a reengineered discharge (RED) program that provided a package of services to decrease readmission rates [7]. This program included six nurse discharge advocates (DA) and a clinical pharmacist. Nurse discharge advocates were responsible for coordinating the discharge plan with the hospital team, creating an after-hospital care plan (AHCP), and providing discharge counseling to patients using teach-back methodology. The AHCP and discharge paperwork were faxed to the patient’s primary care physician. Once a patient was discharged, a clinical pharmacist called the patient to reinforce the discharge plan, review medications with the patient, and address MRPs. If MRPs were found, they were communicated with the primary care provider or DA. Patients provided with this service had lower rates of hospital utilization when compared with usual care.

Reported MRPs per patient ranged from 0.29 to 6.0 [9,11,14,15,16,17]. Our study found a similar number of MRPs (1.8 per patient). By examining the medication discrepancies between the discharge orders, the pharmacy profile, and the patient’s self-report of what they were actually taking, we discovered many sources of potential MRPs. Discrepancies associated with the discharge orders involved use of formulary medications in the same class as medications the patient had been using at home, incomplete or different use instructions, changes in medication strength, missing medications, or continuation of medications used inhouse that did not need to be continued on an outpatient basis. Discrepancies associated with the pharmacy profile involved medications that had been discontinued during the hospitalization that had not been communicated to the pharmacy, new medications that had been started during the hospitalization for which the pharmacy had not yet received a prescription, and changes in medication directions or strengths. Discrepancies associated with patient self-report included not taking medications as directed, not starting new medications, not stopping discontinued medications, and taking two medications from the same therapeutic class.

Although it is clear that community pharmacists are able to identify MRPs and discrepancies post-discharge, the impact of this service has never been quantified in terms of cost avoidance. By using the OutcomesMTM Actuarial Investment Model^^®^^, this pilot project demonstrates that the MRPs resolved by pharmacists provided a total estimated cost avoidance of $92,142 for the 19 patients who received this service, or $4850 per patient. Annualized, it is estimated that 57 BFS patients would be seen, with an annual cost avoidance of $276,450. Based on this cost avoidance and the other findings from this study, BFS has decided to continue this service for its own employees/dependents and to expand this service to any BFS patient discharged from a local regional hospital in the Kansas City area.

Previous studies have identified several barriers to efficient transitions of care, including communication between the hospital and the community pharmacist, identification of patients discharged, contact with patients, lack of time to perform medication reconciliation, incomplete/missing medication list, patient not present during consultation, patient use of multiple pharmacies, and medication reconciliation with multiple providers. The barriers we discovered were lack of discharge paperwork, lack of information about hospitalization (diagnoses), and inability to contact patients who were recently discharged from the hospital. Our pharmacists tried to obtain discharge paperwork either from the patient or requested it from the discharge hospital [6,11,16]. Many hospitals required the completion of a consent form, and it was difficult to have the patient sign and return the form in a timely manner. We also found that the insurance report for identifying patients who were discharged from the hospital did not always include the most pertinent diagnosis. For example, if the patient was admitted to the hospital with pneumonia and had deep vein thrombosis while hospitalized, only the admittance diagnosis would be listed on the report.

We utilized phone and face-to-face meetings at the pharmacy to complete this service. Although the literature has shown that face-to-face interventions are more effective than those delivered via phone [18], the majority of patients in this study preferred to complete this service via phone. The reason for selecting this mode of service was not documented, but telehealth is shown to be a preferred mode of communication for those who are unable to leave home [19]. To provide better quality care, integration of telehealth services into a transitions of care program could be considered.

One limitation of this study was the relatively small sample size. We were able to provide this service to about one half of potentially eligible patients. If this study was performed over a longer period, we would have been able to enroll more patients and obtain a larger sample size. Because this program is being continued, future research can include more patients. Another limitation is that there were two patient outliers, one patient had 12 MRPs identified and resolved, eight of these MRPs were due to adherence and an additional patient had nine MRPs, with eight resulting from non-adherence. Both patients were on nine or more scheduled medications. The literature suggests a linear relationship between the number of MRPs per patient and the number of medications that a patient is taking [20]. This relationship is seen not only in the hospital setting, but also in the ambulatory care and community settings [21,22,23]. In addition, we did not collect data on whether physicians accepted recommendations provided by the pharmacists, or how the pharmacists worked to resolve the MPRs. It would have been meaningful to include data on how many recommendations made to physicians/providers by pharmacists were actually implemented.

We chose residency trained pharmacists to provide this service. However, many other pharmacists should be able to address MRPs and identify discrepancies as this is part of contemporary Doctor of Pharmacy training.

Access to electronic health records would have allowed our pharmacists to address medication discrepancies and MRPs in a timelier manner and may have allowed pharmacists to recognize other MRPs that were not identified. This study shows that there is a need for health care providers to work together to achieve positive health outcomes. Future research should focus on how community pharmacists work with local health care systems to gain access to health records to provide recommendations and counseling to patients.

## 6. Conclusions

Community pharmacists should be a part of the transitions of care team to help prevent medication errors and discrepancies that can lead to increased health care spending. When provided with discharge paperwork, community pharmacists are able to identify and resolve MRPs and discrepancies that might not be recognized by health care providers in the hospital setting. Cost savings associated with this service are sizable. Open communication of medical conditions and discharge paperwork between health care providers is imperative in preventing rising health care costs associated with transitions of care.

## Figures and Tables

**Table 1:** Types of medication-related problems (MRPs) identified during service.

MRP	Frequency
Underuse of medication	24
Dose too low	2
Drug interaction	2
Dose too high	2
Inappropriate administration/technique	2
Needs additional drug therapy	1
Unnecessary drug therapy	1
Suboptimal drug	0
Adverse drug reaction	0
Overuse of medication	0

**Table 2:** Severity levels and frequencies (verified by OutcomesMTM^^®^^ staff).

Severity Level	Number of Events
Level 1: Adherence support	5
Level 2: Reduced drug costs	2
Level 3: Prevented a physician visit	10
Level 4: Prevented additional prescription order	10
Level 5: Prevented emergency room visit	4
Level 6: Prevented hospital admission	3
Level 7: Prevented a life-threatening situation	0

**Table 3:** Correlations among variables.

	Number of Medications with Discrepancies	Number of MRPs
Number of scheduled prescription medications	0.825 **	0.697 **
Number of medications with discrepancies	1	0.701 **
Number of discrepancies between discharge paperwork and pharmacy dispensing profile	0.854 **	0.849 **
Number of discrepancies between discharge paperwork and pharmacy dispensing profile	0.974 **	0.610 *
Number of discrepancies between pharmacy dispensing profile and patient reported medication list	0.422	0.531 *
Number of disease states	0.823 **	0.7 **
Number of MRPs	0.701 **	1
Medication reconciliation before discharge	−0.667 **	−0.765 **

MRPs = medication-related problems. * Correlation is significant at the 0.05 level (2-tailed); ** Correlation is significant at the 0.01 level (2-tailed).

## Supplementary Materials

The following are available online at https://www.mdpi.com/2226-4787/7/2/51/s1, Modified CMR Worksheet and OutcomesMTM^^®^^ Encounter Worksheet.
