# Supplementary material for: A Study to Identify Medication-Related Problems and Associated Cost Avoidance by Community Pharmacists during a Comprehensive Medication Review in Patients One Week Post Hospitalization
Source: Pharmacy (Basel). 2019 May 29;7(2):51. doi: 10.3390/pharmacy7020051 (PMC6630417; doi:10.3390/pharmacy7020051)
Supplement: Supplementary file 1 [file pharmacy-07-00051-s001.zip › pharmacy-505894-final-supplementary materials/Modified CMR Worksheet.pdf]

Modified CMR Worksheet

Patient Name: ______________________________________ DOB: ___________________

Address: ____

Is the patient cognitively impaired? Yes / No

Is the CMR with the patient? Yes / No

Phone #: _______________________

Assistance by (name): _____________________ Relationship: ____________________

Did the patient receive a medication reconciliation before discharge? Yes / No

By whom: □ Pharmacist □ Physician □ Nurse □ Other: ___________

Date of discharge: _____________________

Hospital diagnosis: ____________________

Date of CMR: __________________________

Med List & MAP mailed: ________________

Follow-up completed: _________________

Pharmacist: ___________________________

Location: □ In store □ At home □ By phone

Patient's pharmacy: ____________________

Medication Profile

❶ Medical conditions

- ☐ Alzheimer's
- ☐ Anemia
- ☐ Anticoagulation
- ☐ Asthma
- ☐ Autoimmune Disorders
- ☐ Benign Prostatic Hyperplasia
- ☐ Bipolar Disorder
- ☐ Depression
- ☐ Diabetes
- ☐ Dyslipidemia
- ☐ GI/Reflux/Ulcer
- ☐ Hepatitis C
- ☐ HIV/AIDS
- ☐ Hypertension
- ☐ Multiple Sclerosis
- ☐ Neurologic Disorders
- ☐ Osteoarthritis
- ☐ Osteoporosis
- ☐ Parkinson's Disease
- ☐ Rheumatoid Arthritis
- ☐ Schizophrenia
- ☐ Severe Hematologic Disorder
- ☐ Stroke
- ☐ Surgery ____________

❷ Drug Allergies or Intolerances

☐ No known drug allergies

Medication	Reaction

APPENDIX A. Modified CMR Worksheet

❸ Physicians and Hospitalists (name, phone number, type of physician)

**Name**	**Type of Physician**	**Phone number**	**Hospital/ Clinic**

❹ Medications

	**What Patient is Actually Taking (From Patient Interview)**			**Prescribed Post Discharge (From Discharge Paperwork)**			**Before Hospital (From Pharmacy Profile)**		
Drug Name	Strength	Regimen	Prescriber/ Indication	Strength	Regimen	Prescriber/ Indication	Strength	Regimen	Prescriber/ Indication

APPENDIX A. Modified CMR Worksheet

Pharmacist's Notes:

APPENDIX A. Modified CMR Worksheet

❺ Detailed Medication Action Plan for Pharmacists

Medication	Description of the problem	How it was resolved

APPENDIX A. Modified CMR Worksheet

Medication Action Plan

Patient Name: ____

Pharmacist Name: ____

Date of visit with pharmacist: ________________________________

Pharmacist's Phone Number: _______________________________

Medication	Description of the problem	What I should do

Please feel free to call if you have any questions or concerns!
