# Supplementary material for: A Study to Identify Medication-Related Problems and Associated Cost Avoidance by Community Pharmacists during a Comprehensive Medication Review in Patients One Week Post Hospitalization
Source: Pharmacy (Basel). 2019 May 29;7(2):51. doi: 10.3390/pharmacy7020051 (PMC6630417; doi:10.3390/pharmacy7020051)
Supplement: Supplementary file 1 [file pharmacy-07-00051-s001.zip › pharmacy-505894-final-supplementary materials/OutcomesMTM® Encounter Worksheet.pdf]

Encounter**Worksheet**

OUTCOMESMTM™
The Face-to-Face Difference

PRESCRIPTION INFORMATION

Patient Info/Rx Info

Patient ID Number	Gender	Date of Birth
	M F	Y Y Y Y M M D D

Patient Last Name

Patient First Name

New Medication

New Rx Date	New Rx Number	Metric Qty.	Days Supply
M M D D Y Y Y Y			

New Rx National Drug Code – Labeler	Product	Pkg	New Rx Prescriber ID

MONITORING

	Attempts	Appointment
1	____/____/____ @ ____:____ AM/PM	_______ / _______ / ___________
2	____/____/____ @ ____:____ AM/PM	_______ : _______ AM / PM
3	____/____/____ @ ____:____ AM/PM	(_______) _______ - __________

Current Medication

Current Rx Date	Current Rx Number	Metric Qty.	Days Supply
M M D D Y Y Y Y			

Current Rx National Drug Code – Labeler	Product	Pkg	Current Rx Prescriber ID

ENCOUNTER DOCUMENTATION

Date of Encounter/Follow-up ________ Claim Number ________

I. Indication For Service (REASON)		II. Service Provided (ACTION)		III. Outcome of Service (RESULT)	
CMR - Complex Drug Therapy	100	Comprehensive Med Review (CMR)	200	CMR - Drug Therapy Problems Identified	300
				CMR - No Drug Therapy Problems Identified	301
				Patient Refused	380
Cost Effective Alternative	105	Prescriber Consultation	205	Initiated Cost Effective Drug	305
				Prescriber Refused Recommendation	375
				3 Attempts Unable to Reach Prescriber	378
Cost Effective Alternative	105	Patient Consultation	215	Patient Refused	380
New/Changed Prescription Therapy	110	Patient Education and Monitoring	210	Therapy Success *(Resolved/Stable)*	310
New/Changed OTC Therapy	117			Therapy Failure *(Unresolved/Worse)*	320
				Patient Refused	380

Patient Education: *Initial consultation date:* MM / DD / YYYY

- ❒ Name of Drug
- ❒ Therapeutic Class
- ❒ Directions for Use
- ❒ Side Effects/Warnings
- ❒ Storage Requirements
- ❒ Missed Dose Actions
- ❒ Written Material
- ❒ Set Monitoring Appointment

Monitoring: *Follow-up date:* MM / DD / YYYY

Is the patient satisfied with the therapy?	❒ Yes	❒ No
Did the symptoms improve?	❒ Yes	❒ No
Were any adverse reactions reported?	❒ Yes	❒ No
Is the patient adherent with therapy?	❒ Yes	❒ No

DRUG THERAPY PROBLEM DETECTED:		DRUG THERAPY PROBLEM RESOLVED:	
**INDICATIONS**		**INDICATIONS**	
Needs Drug Therapy	120	Initiated New Therapy	330
Unnecessary Prescription Therapy	125	Discontinued Therapy	335
**EFFICACY**		**EFFICACY**	
Suboptimal Drug	130	Changed Drug	340
Dose Too Low	135	Increased Dose	345
**SAFETY**		**SAFETY**	
Adverse Drug Reaction	140	Decreased Dose	355
Drug Interaction	145	Prescriber Refused Recommendation	375
Dose Too High	150	3 Attempts Unable to Reach Prescriber	378

Prescriber Consultation 205

ADHERENCE		Patient Adherence Consultation	215	ADHERENCE	
Overuse of Medication	155	❒ Determine Barriers to Adherence		Altered Adherence	360
Underuse of Medication	160	*Date non-adherence was identified:* MM / DD / YYYY		Altered Administration or Technique	365
Inappropriate Admin/Technique	165	❒ Education		Patient Refused	380
		❒ Set Monitoring Appointment		3 Attempts Unable to Reach Patient	379
		Date non-adherence was resolved: MM / DD / YYYY			

IV. Severity Level

❒ **Level 1** Adherence Support	❒ **Level 4** Prevented Additional Prescription Order	❒ **Level 7** Prevented a Life Threatening Situation
❒ **Level 2** Reduced Drug Costs	❒ **Level 5** Prevented Emergency Room Visit	
❒ **Level 3** Prevented a Physician Visit	❒ **Level 6** Prevented Hospital Admission	

V. Severity Level Rationale & Additional Notes

Pharmacist Name	NCPDP/NABP	RPh Initials

Copyright © OutcomesMTM™ All Rights Reserved
